# Sexual dimorphism in dinosaurs

**DOI:** 10.7554/eLife.89158

**Published:** 2023-06-14

**Authors:** Stella A Ludwig, Roy E Smith, Nizar Ibrahim

**Affiliations:** 1 School of the Environment, Geography and Geosciences, University of Portsmouth Portsmouth United Kingdom

**Keywords:** intraspecific variability, dinosaurs, 3D geometric morphometrics, sexual dimorphism, limb bones, palaeontology, Other

## Abstract

Studying fossils from a mass-mortality event reveals evidence for sexual dimorphism and, unusually, equal numbers of males and females in a herd of dinosaurs.

**Related research article** Pintore R, Cornette R, Houssaye A, Allain R. 2023. Femora from an exceptionally large population of coeval ornithomimosaurs yield evidence of sexual dimorphism in extinct theropod dinosaurs. *eLife* **12**:e83413. doi: 10.7554/eLife.83413.

Sexual dimorphism – sex-specific differences in morphology and appearance – can be observed in many animals and is most obvious when it involves external and soft-tissue features, such as reproductive organs or brightly coloured feathers. In many cases, these features are accompanied by corresponding variations in the skeleton which can be subtle, but still sufficient to confidently tell the sexes apart. However, identifying differences in extinct animals remains a significant challenge, with a clear-cut example of sexual dimorphism in dinosaurs proving particularly elusive.

The vast majority of dinosaur species have been identified from incomplete and fragmentary fossilised remains, and even near-complete skeletons rarely contain preserved soft tissues. In the past, researchers have used a wide range of skeletal features – including tail anatomy, overall robustness, and skull ornamentation – as evidence of sexual dimorphism in dinosaurs (see for instance Chapman et al., 1997; Barden and Maidment, 2011; Padian and Horner, 2011; Mallon, 2017; Saitta et al., 2020). However, over millions of years, the bones have undergone significant and variable stresses, altering their shape in unique ways, and making it difficult to tell whether differences are due to modifications during fossilisation, sexual dimorphism, or individual variation within a species.

Although it might be assumed that a dinosaur skeleton found sitting on a clutch of eggs would be female, evidence from fossil egg clutches and modern birds suggests that male dinosaurs likely had a significant role in parental care in some species, resulting in such skeletons being assigned to both sexes in the past (see for example Norell et al., 1995; Grellet-Tinner et al., 2006; Martill et al., 1996; Varricchio et al., 2008, Yang et al., 2019). Additionally, the presence of medullary bone – a layer of material within bones which helps females to build an eggshell – has been used to sex dinosaurs. However, this material is only present when females are preparing to lay eggs, so it cannot reliably identify females at other times (Prondvai, 2017). Furthermore, skeletons are often found in different locations and the preserved animals may have lived several thousand or even millions of years apart, making it impossible to tell whether any variations in the skeleton are due to regional, temporal, individual, age-related or sex-related differences.

Therefore, demonstrating sexual dimorphism in a group of dinosaurs requires: i) a large sample size to allow for individual variation; ii) a method for identifying sexual dimorphism that is informed by the variation displayed by animals still in existence today. Now, in eLife, Romain Pintore, Raphaël Cornette, Alexandra Houssaye and Ronan Allain – who are based at the CNRS/Muséum national d’Histoire naturelle – report that the thigh bones of dinosaurs called ornithomimosaurs display several features that vary between the sexes (Pintore et al., 2023).

Pintore et al. studied a herd of at least 61 ornithomimosaurs deposited in a fossil bed in Angeac-Charente in the south-west of France. The herd died in a mass-mortality event in the Berriasian (Early Cretaceous) period – this presented the researchers with the opportunity to compare the fossilised remains of a large number of dinosaurs from the same place and time. Such opportunities are rare in palaeontology.

The researchers performed 3D geometric morphometric analysis – which can quantify subtle variations in biological shape – of the best-preserved hindlimb bones. The results showed that the thigh bones of the dinosaurs displayed subtle differences in shape that were independent of the size of the bone, with the most important variation being in the curvature of the femur (Figure 1). Given that similar differences in curvature have been observed between the sexes among the remaining archosaurs – such as birds and crocodiles – Pintore et al. attributed them to sexual dimorphism in ornithomimosaurs.

Having identified features which allowed them to differentiate between the sexes, Pintore et al. counted equal numbers of males and females in the Angeac-Charente ornithomimosaur herd. This differs from populations of some remaining archosaurs, whose initial equal sex ratio becomes skewed towards females in sub-adult and adult populations (Woodward and Murray, 1993; Magige, 2012; González et al., 2019; Prokopenko et al., 2021). Future work should investigate growth-related variations within the ornithomimosaur sample and determine the sex corresponding to each femur type. Such investigations could clarify whether sex ratios vary among ornithomimosaurs or whether they maintain an equal sex ratio across all age groups within the herd.

The findings are the first to examine sexual dimorphism in a group of dinosaurs that lived and died together, and they have significant implications for our understanding of dinosaur sex differences, behaviour and group dynamics. The work emphasises the importance of describing size-independent dimorphism in modern animals, where sex can be more easily determined, to aid interpretation of morphological differences in extinct species. Understanding how sex impacts the shape and size of extinct species also has broader implications for evolutionary mechanisms and, ultimately, our understanding of ancient ecosystems.

**Figure 1.**
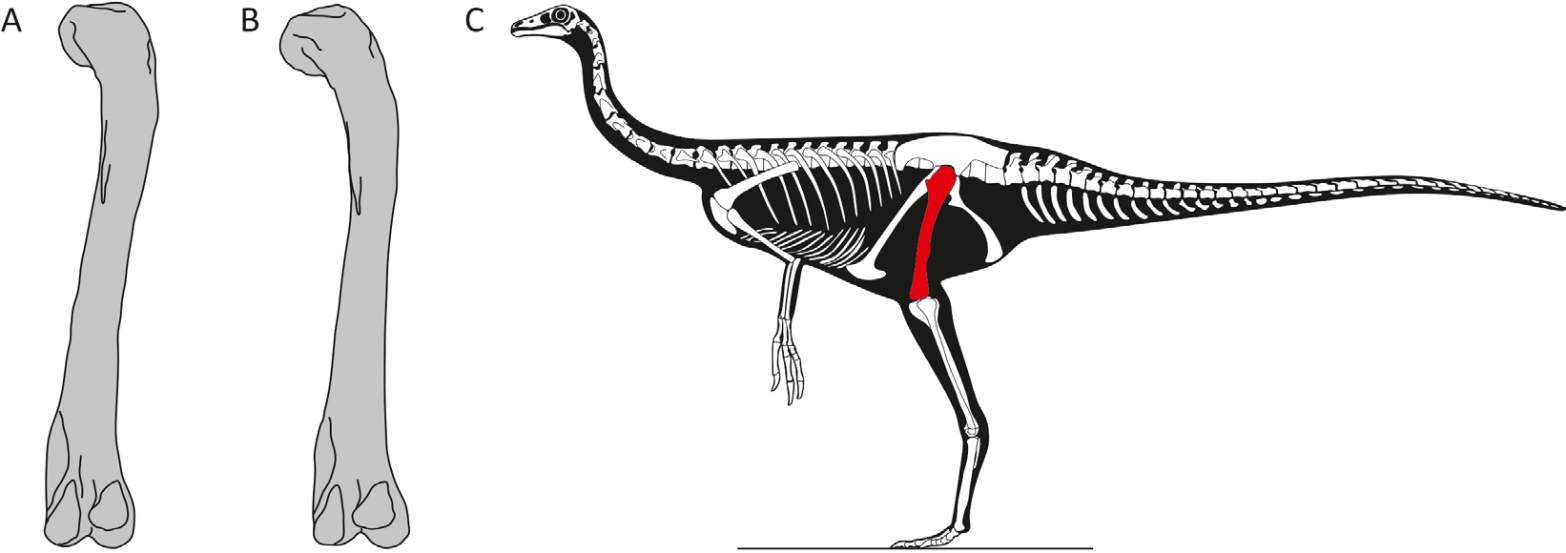
Sexual dimorphism in ornithomimosaurs. (**A–B**) Pintore et al. identified subtle differences in the curvature and shape of the femur in fossils of ornithomimosaurs from the Angeac-Charente fossil site in France. Further work is needed to determine the sex corresponding to each type of femur. (**C**) Skeletal outline of an ornithomimosaur based upon *Struthiomimus* from Longrich, 2008; fig. 2, with the femur highlighted in red.
